# Metabolomics in Animal Models of Bronchial Asthma and Its Translational Importance for Clinics

**DOI:** 10.3390/ijms25010459

**Published:** 2023-12-29

**Authors:** Romana Barosova, Eva Baranovicova, Juliana Hanusrichterova, Daniela Mokra

**Affiliations:** 1 Department of Physiology, Jessenius Faculty of Medicine in Martin, Comenius University in Bratislava, 03601 Martin, Slovakia; palova31@uniba.sk (R.B.); topercerova4@uniba.sk (J.H.); 2 Biomedical Center Martin, Jessenius Faculty of Medicine in Martin, Comenius University in Bratislava, 03601 Martin, Slovakia; eva.baranovicova@uniba.sk

**Keywords:** metabolomics, bronchial asthma, animal model

## Abstract

Bronchial asthma is an extremely heterogenous chronic respiratory disorder with several distinct endotypes and phenotypes. These subtypes differ not only in the pathophysiological changes and/or clinical features but also in their response to the treatment. Therefore, precise diagnostics represent a fundamental condition for effective therapy. In the diagnostic process, metabolomic approaches have been increasingly used, providing detailed information on the metabolic alterations associated with human asthma. Further information is brought by metabolomic analysis of samples obtained from animal models. This article summarizes the current knowledge on metabolomic changes in human and animal studies of asthma and reveals that alterations in lipid metabolism, amino acid metabolism, purine metabolism, glycolysis and the tricarboxylic acid cycle found in the animal studies resemble, to a large extent, the changes found in human patients with asthma. The findings indicate that, despite the limitations of animal modeling in asthma, pre-clinical testing and metabolomic analysis of animal samples may, together with metabolomic analysis of human samples, contribute to a novel way of personalized treatment of asthma patients.

## 1. Introduction

Bronchial asthma is an extremely heterogenous chronic respiratory disorder that develops in all age groups of patients in several distinct endotypes and phenotypes. These subtypes differ not only in the pathophysiological changes and/or clinical features but also in their response to the treatment. Although enormous efforts have been devoted to understanding asthma pathophysiology, more detailed asthma phenotyping is required for precise diagnosis and personalized therapy. In the diagnostic process, metabolomic approaches have been increasingly used, providing detailed information on the metabolic alterations associated with human asthma. Further information is brought by metabolomic analysis of samples obtained from animal models. Thus, metabolomics and other “omics” platforms are starting to be a standard approach in order to gain more detailed understanding of the disease and help to evaluate precisely an effect of the treatment. This article summarizes the current knowledge on metabolomic changes in human and animal studies of asthma and discusses the translational potential of pre-clinical research for clinics.

## 2. Bronchial Asthma

### 2.1. Features of Bronchial Asthma

Bronchial asthma is a chronic respiratory disease characterized by airway inflammation, bronchial hyperresponsiveness, increased mucus production, and variable degree of airflow limitation. These pathophysiological changes result in typical clinical signs such as intermittent wheeze, cough or chest tightness due to reversible airway obstruction [1]. Although the number of deaths due to asthma has reduced greatly over the past two decades, the trend of increasing prevalence of asthma has continued over the last few years. It is recognized that most cases of adult asthma are primarily developed in childhood [2]. Considering diverse underlying mechanisms, asthma seems to be dependent on both genetic predisposition and environmental factors [1], with distinct proposed risk factors for each endotype of asthma [2].

Based on the pathomechanisms, several endotypes of asthma, such as eosinophilic, non-eosinophilic, mixed granulocytic asthma or paucigranulocytic asthma, can be distinguished according to the type of inflammation, contribution of inflammatory cells, extent of mucus production, increase in airway smooth muscle mass etc. [1,3]. Nevertheless, most frequently, asthma has been divided into two basic endotypes: type-2 (T2 or T-helper (Th)2)-high endotype with type-2 immune response and recruitment of eosinophils; and type-2 (T2 or Th2)-low or non-type-2 endotype that is non-eosinophilic and in which inflammation is mediated via activation of Th1 and/or Th17 cells [3]. In addition, several asthma phenotypes may be distinguished according to clinical features and time of initial clinical presentation [1,3]. T2-high asthma includes three clinical phenotypes, i.e., early-onset atopic asthma, late-onset asthma, and aspirin-exacerbated respiratory disease. The best understood subtype is allergic or atopic phenotype regulated by CD4-positive T-cells producing Th2-type cytokines such as interleukin (IL)-4, IL-5, IL-9, IL-13 and granulocyte-macrophage colony-stimulating factor (GM-CSF) accompanied by the production of allergen-specific immunoglobulin (Ig)E antibodies by B-cells. Atopy was identified in approximately 80% of early-onset asthma and 50% of late-onset eosinophilic asthma [3,4]. On the other hand, obesity-associated asthma, smoking-associated asthma and very late-onset asthma phenotypes, characterized by onset after the 5th life decade, are types of T2-low asthma [3,5]. According to Severe Asthma Research Program (SARP) cluster analysis, early-onset asthma is highly associated with allergies, while patients with the later-onset disease suffer from more severe airflow limitation and less allergic manifestation [6].

Identification of specific biomarkers of asthma endotypes and phenotypes is of the utmost importance in order to provide adequate treatment, as different endotypes/phenotypes may respond differentially to a given therapy [7]. For instance, the atopic early-onset asthma phenotype is a typical representative of allergic eosinophilic asthma that responds well to corticosteroids (CS). However, the obesity-associated asthma phenotype belonging to non-eosinophilic asthma is CS-resistant [7,8]. Thus, identification of type of predominant inflammation and related endotype/phenotype may suggest which kind of treatment may be effective. In eosinophilic asthma, inhaled CS (ICS) and/or oral CS represent a treatment of choice that may be combined with bronchodilators, theophylline, or biological treatment such as anti-IgE, anti-IL-5/IL-5R, anti-IL-4R, anti-IL-13, anti-thymic stromal lymphopoietin (TSLP) or anti-leukotrienes. In neutrophilic asthma, the effect of CS is limited and other treatments including macrolides, long-acting muscarinic antagonists (LAMA), bronchodilators, theophylline, phosphodiesterase 4 inhibitor roflumilast, statins or biological treatments such as anti-tumor necrosis factor (TNF)α, anti-IL-17, or chemokine (C-X-C motif) receptor (CXCR2) antagonists can be used. In paucigranulocytic asthma, where eosinophilic and/or neutrophilic inflammation are absent but airway hyperresponsiveness dominates, prospective approaches are limited to bronchial thermoplasty, LAMA bronchodilators or mast-cell directed therapy [7,9,10].

### 2.2. Diagnostic Tools in Asthma

Traditional asthma criteria are based on a set of features and clinical characteristics such as wheeze, shortness of breath, chest tightness, and cough and on variable expiratory airflow limitation. In addition, the diagnostic algorithm includes sequential tests: spirometric tests of airway obstruction, bronchodilator reversibility, verification of eosinophilic airway inflammation by measurement of IgE, sputum and blood eosinophils, and periostin, fractional exhaled nitric oxide (FeNO), and airflow variability and/or bronchial challenge (airway provocation) test or exercise challenge test [1]. However, these measurements do not adequately reflect the level of inflammation and bronchoconstriction that is responsible for the fact that asthma is underdiagnosed in many patients [11]. Over the years, it has been recognized that asthma consists of different endotypes/phenotypes [12] that were later systematically ordered after hierarchical cluster analysis of population in SARP [6] and the UK Leicester adult cohorts [13]. Nevertheless, despite recognition of the endotypes/phenotypes of asthma owing distinct features and requiring different therapeutic strategies in terms of personalized treatment, management of asthma still continues according to Global Initiative for Asthma (GINA) guidelines on the basis of the severity of the condition, with drugs added only on the basis of symptom control achieved [11]. Thus, in order to deliver personalized medicine for bronchial asthma, novel diagnostic methods should be used and additional biomarkers recognized [14].

### 2.3. Advances of “Omics” in Asthma

Over the last few years, researchers have been focusing on the holistic view of the molecular pathomechanisms underlying various diseases including asthma. Advanced analytical methods allow the performing of large-scale and comprehensive analyses named “omics”, greatly contributing to the integrated view of biological systems. Metabolomics has been labeled as one of the new “omics”, joining genomics, transcriptomics, and proteomics as a science employed toward the understanding of global systems biology. Advances in “omics” including metabolomics may help to elucidate specific biochemical perturbations in asthma [14,15,16,17].

Metabolomic analyses of exhaled breath condensate (EBC) and FeNO have already been well-established in the diagnostics of asthma. FeNO brings valuable information on exhaled NO levels associated with eosinophilic inflammation and metabolites of lipid peroxidation [14,18]. Analysis of volatile organic compounds (VOCs) in the exhaled air represents a fast and easy-to-use method for detection and identification of several compounds, including alkanes, which may serve as potential biomarkers of asthma [19,20]. Nowadays, the identification and validation of relevant biomarkers or predictors of asthma (especially of pediatric, severe, and CS-resistant asthma) has emerged as the highest priority across leading experts, researchers, and clinicians [21]. Multi-omics approaches may be particularly helpful in the pediatric population, where the “gold standard” diagnostic tests (e.g., spirometry) may be difficult to perform. Moreover, these novel approaches may be useful in early asthma that is hardly revealed using standard biomarkers and methods [22].

## 3. Metabolomics in Asthma

As mentioned before, asthma covers different endotypes/phenotypes with different inflammatory characteristics and clinical features. The complexity of this disease has already been demonstrated on genomic, transcriptomic, proteomic, and metabolomic levels in a lot of studies. The last mentioned, the metabolomic approach, profits from advantages such as great sensitivity and reproducibility, a knowledge basis that comes from years of biochemical research, and the relatively small size of the recognized number of endogenous metabolites/molecules related to the number of genes, RNA species, or proteins [23]. Nevertheless, the metabolites are not only the intermediates or end products of biochemical pathways. These small molecules (Mm < 1500 Da) also participate in a variety of processes, including energy metabolism, and act as neurotransmitters in signaling pathways, modulators of immune response and protein activity, or signalers of epigenetic changes. In addition, single metabolites or their clusters are of high potential in discriminatory analysis in the search for potential disease biomarkers [24].

Metabolomic strategies have been divided into two basic approaches, untargeted metabolomics (known as metabolic fingerprinting) and targeted metabolomics (or metabolic profiling). Untargeted metabolomics is the comprehensive analysis of all the measurable analytes in a sample, whilst targeted metabolomics is the measurement of defined groups of chemically characterized and biochemically annotated metabolites [25].

In contrast to genetics and transcriptomics, metabolomics has no single technique for comprehensive profiling of the entire metabolome because of intrinsic variation in the physical-chemical properties of different metabolite classes, from small polar volatiles to large hydrophobic lipids. Metabolites can be measured by various analytical techniques, but primarily by one-dimensional (1D) proton (H) nuclear magnetic resonance (NMR) or mass spectroscopy (MS), preceded by either liquid chromatography (LC) or gas chromatography (GC) [24,26]. In principle, the NMR-based approach is used for detection of a broad spectrum of metabolites and searching potential biomarkers, while more sensitive MS-based analyses are used when limited numbers of metabolites in low concentrations should be measured [26]. Changes in metabolites can be detected in urine, blood/plasma/serum, EBC, bronchoalveolar lavage fluid (BALF), stools, tissue samples, etc. [16,17,23]. In addition, analytical methods are combined with sophisticated bioinformatic systems and statistical methods in order to describe the metabolome of a given biological system [26,27].

Metabolomic studies may reflect local changes as well as systematic alterations or dysregulations in the organism [16]. In asthma, alteration in metabolome due to disordered metabolic pathways (oxidation–reduction imbalance, disturbances of energy, lipid, and/or amino acid metabolisms) may mirror the changes associated with airway inflammation, airway obstruction, and mucus production [16] that may correlate with standard diagnostic parameters (functional lung tests, IgE levels, FeNO, etc.) [28]. In addition, metabolomics can reveal valuable biomarkers differentiating asthma patients from healthy individuals [15], distinguish the extent of asthma severity [29], help to identify asthma endotypes/phenotypes [30], or evaluate responses to given treatments [31,32].

Recently, several excellent articles have discussed changes in metabolome in relation to asthma [15,16,23,33,34]. In addition to analyses of patient samples, there is an increasing number of animal studies which may bring more light into the pathophysiology and appropriate therapy of asthma. The most frequently used models of allergic asthma utilize ovalbumin (OVA) or house dust mite (HDM) sensitization to elicit eosinophil-mediated inflammatory changes, while instillation of fine particulate matter (PM2.5) may evoke activation of both Th2- and Th1/Th17-mediated immune responses. In spite of the fact that bronchial asthma is observed exclusively in humans and, therefore, no animal model can fully mimic the asthma in human [35], animal models of asthma are valuable for understanding pathophysiological mechanisms underlying the disease and have been widely used to test novel therapeutics. Subsequent sections of this article summarize basic metabolomic data collected from clinical and experimental studies demonstrating the differences between asthmatic vs. healthy individuals, obesity-associated asthmatics vs. healthy individuals, differences according to sex and age, and the effects of lifestyle, diet and microbiome as well as the effects of delivered treatments.

### 3.1. Metabolomics Data for Comparison between Asthma vs. Healthy Individuals

#### 3.1.1. Human Studies

In asthma patients, the most prominent changes have been observed for lipid metabolism. Lipids represent the majority of the biological molecules in the blood plasma and about half of the mass of the cell membrane [36]. They may be classified into several categories with distinct biological functions in the body, such as energy conversion and storing, transport of substances, signal transduction, cell development and differentiation or regulation of cell death and autophagy [36,37]. In addition, lipids are the major component of pulmonary surfactant, a vitally important mixture reducing the surface tension at the air–liquid interface in the alveoli [38]. Nevertheless, lipids also play a fundamental role in inflammation and oxidative stress, contributing to the development of chronic respiratory diseases including asthma [29,39,40]. In asthma patients, numerous studies have shown serious alterations in lipid metabolism, particularly in glycerophospholipid metabolism and fatty acid metabolism. For instance, significantly increased phosphatidylethanolamine (PE) was found in BALF [41] and serum of asthmatic patients [39]. Phosphatidylglycerol (PG) may inhibit pro-inflammatory cytokine production; however, hydrolysis of PG may enhance Th2-mediated inflammation [42]. On the other hand, decreased levels of lysophosphatidylcholine (lysoPC) may be associated with more severe asthma [39,43,44]. Sphingomyelin (SM) may also change in asthma patients [39,45], while a decrease in sphingolipid synthesis may be related to airway smooth muscle contraction [46]. Asthma development is also associated with intake of lipids and obesity, as fatty oxidation is increased in asthma [47]. Poly-unsaturated fatty acids (PUFA) (or omega-3 fatty acids) such as docosahexaenoic acid (DHA) and eicosapentaenoic acid (EPA) may exert protective effects in asthma as they downregulate airway eosinophilic inflammation and promote the resolution of inflammation [48,49], while short-chain fatty acids such as butyric acid can inhibit airway hyperreactivity [50]. Bian et al. showed significant changes in hydroxyeicosatetraenoic acids (HETEs), hydroperoxyeicosatetraenoic acids (HPETEs), EPA, ursodeoxycholic acid, deoxycholic acid, isodeoxycholic acid, palmitic acid, 2-lauroleic acid and lauric acid, as well as in several prostaglandins in asthma patients compared with controls [51]. In addition, several triglycerides (TG) may increase in asthma [39,52], while TG (16:0/16:0/18:1) together with PE (20:0/18:1) showed a relation to IgE levels in asthmatic patients [39]. TG are closely linked with arachidonic acid metabolism, leading to the production of various products including prostaglandins, thromboxanes and leukotrienes that also play an important role in the pathogenesis of asthma [51,53,54].

The shift in lipid metabolites may correlate with asthma severity. Loureiro et al. found that metabolites of lipid peroxidation comprising 34 aliphatic alkanes and aldehydes measured in urine samples were closely related to disease severity, alteration of lung function and eosinophilic inflammation [55]. In agreement with this study, Reinke et al. identified 66 metabolites, from which 15 were significantly altered with asthma. In addition, the authors found that the metabolic shifts in mild asthma, relative to controls, were linked with exogenous metabolites (i.e., elevated levels of dietary lipids such as linoleic acid, oleic acid, α-linolenic acid and linoleic acid oxidation products) [29], confirming the previous findings that linoleic acid and its oxidation products likely contribute to Th2 differentiation in asthma [56]. On the other hand, moderate and severe asthma was associated with increases in other metabolites, e.g., oleoylethanolamide, sphingosine-1-phosphate, and N-palmitoyltaurine, involved in activating the transient receptor potential vanilloid type 1 (TRPV1) receptors [29] which act as Ca^2+^ ion channels in sensory nerves, and contribute to chemotaxis, bronchoconstriction, mucous secretion, airway irritation and the urge to cough [57].

In addition, asthma is associated with alterations in amino acid metabolism, as the most prominent changes have been observed for metabolisms of arginine, tyrosine, valine, tryptophan, and taurine. Arginine is a substrate for arginase-2 (ARG2) but also for inducible nitric oxide synthase (iNOS), which is linked to airway inflammation. Arginine metabolism as a key driver of Th2-mediated airway inflammation is increased in asthmatics compared with healthy humans [58]. Lower levels of L-arginine in asthmatic patients [59,60,61] could be explained by higher expression of arginase in the respiratory epithelium and in the inflammatory cells [60]. Asthmatics with high FeNO had higher expression of iNOS and ARG2 in the airway, which was associated with more severe asthma [62]. The arginine metabolism seems to be a critical modulator of severity of inflammation and remodeling in both eosinophilic and neutrophilic asthma, while arginine metabolism via ARG2 suppresses inflammation and, via iNOS, promotes airway inflammation [63].

The amino acid valine plays an important role in muscle metabolism. Its changed levels were found in asthmatic children [64,65,66] and in asthma–COPD overlap syndrome [67]. Similarly, tyrosine, which was recognized as a potentially protective factor against allergic airway inflammation [68], was identified as one of the metabolites associated with lowly sensitized asthma in children [66]. On the other hand, the metabolic pathway of tryptophan and production of 5-hydroxytryptamine contributes to development of allergic inflammation [69]. Asthma is also associated with changes in plasma levels of taurine, a ubiquitous amino acid with anti-oxidant and anti-inflammatory effects, that may be found especially in polymorphonuclears and tissues exposed to oxidative stress [70]. Comhair et al. found significantly higher plasma levels of taurine in asthmatics; however, taurine levels did not correlate with severity of asthma nor FeNO. Elevated concentrations of substances which are included in the bile acid production pathway, such as taurine, lathosterol, and bile acids (taurocholate and glycodeoxycholate), in asthmatics suggest a role of NO-associated taurine transport and bile acid metabolism in the pathogenesis of asthma [54]. Significantly increased amino acids taurine and asparagine were previously determined in the BALF of the asthmatic patients [41]. It is supposed that higher NO produced in asthmatic airways may impair the taurine transporter [71]. Increased plasma taurine then causes higher formation of bile acids, which is a major form of taurine elimination [54]. Additional alterations in amino acid profile were also identified in asthmatic children compared with non-asthmatic controls; however, some changes were distinct from the findings in adult patients, as the authors found decreased serum concentration of taurine, L-valine, and DL-β-aminoisobutyric acid, and increased levels of ƴ-amino-n-butyric acid and L-arginine [64]. Asthmatics also had higher levels of substances related to the glutamate–glutamine cycle, glutamylphenylalanine and glutamyltyrosine, as compared with the healthy controls [54,59,72]. Further changes were observed for the methionine–glutathione cycle [59,65].

In addition to the above-mentioned metabolic pathways, the purine metabolism is also altered in asthma. Uric acid, a product of the purine metabolism, enhances Th2-mediated immune response [71]. Increased levels of uric acid and/or hypoxanthine were found by Liang et al. [72] and Tao et al. [73], confirming the role of purine metabolism alterations in asthma.

Additional metabolic changes in asthmatic patients are associated with altered glycolysis and the tricarboxylic acid (TCA) cycle. Glycolysis converts glucose to pyruvate in 10 steps. Under aerobic conditions, pyruvate is transported to mitochondria and converted to acetylCoA, which enters the TCA cycle, leading to the production of energy in the form of adenosine triphosphate (ATP). As regulation of the TCA cycle depends on the availability of substrates, alteration of energy metabolism in asthma leads to changes in glucose, pyruvate, citrate, succinate, malate, etc. [59,65,67,74,75]. Under stress and in anaerobic conditions, pyruvate may change to lactate, representing an alternative source of energy. In asthma, levels of lactate may be increased [55,76,77].

As mentioned above, numerous studies have demonstrated that asthma is associated with alterations in several metabolic pathways. Moreover, some authors detected the differences between metabolic changes in the eosinophilic and non-eosinophilic asthma that may help to identify the individual endotypes or phenotypes of asthma. For instance, in the study by Comhair et al., the plasma metabolomes of severe asthmatics (based on the use of high doses of CS treatment), asthmatics with high FeNO (a biomarker of predominantly Th2-high or eosinophilic airway inflammation), and healthy controls were evaluated. Severe asthmatics showed biochemical changes related to changes in steroid metabolism and amino acid/protein metabolism (indicated e.g., by increased β-alanine). On the other hand, asthmatics with high FeNO had elevated levels of branched-chain amino acids (leucine, isoleucine, valine) and bile acids (glycocholate, cholate) [54]. In a more recent study, metabolomic analysis revealed changes in glycerophospholipid, retinol, sphingolipid, galactose, inositol phosphate and linoleic acid metabolisms between patients with allergic asthma and healthy controls. On the other hand, changes in the glycerophospholipid, sphingolipid, galactose arachidonic acid, inositol phosphate, starch and sucrose and linoleic acid metabolisms were identified between non-allergic asthma and healthy controls. Further important differences in the glycerophospholipid, retinol, sphingolipid, galactose and inositol phosphate metabolisms were observed for comparison between the eosinophilic and non-eosinophilic asthma [44].

#### 3.1.2. Animal Studies

In the last two decades, several experimental studies evaluated metabolomic changes associated with OVA sensitization. For instance, Saude et al. found significant shifts in metabolites, e.g., decreased levels of 2-hydroxyisobutyrate, 3-hydroxybutyrate, 3-methyladipate, tyrosine, glucose and creatine in urine samples of OVA-sensitized female guinea pigs compared with controls [78]. In OVA-sensitized female BALB/c mice, changes in six metabolic pathways (dodecanoic acid, myristic acid, phytosphingosine, sphinganine, inosine and taurocholic acid) were found, from which the purine metabolism was the most prominently disturbed, as well as the metabolites of purine metabolism, uric acid and inosine. The authors supposed that inflammatory responses in allergic asthma are responsible for decreased plasma uric acid due to inhibition of the activity of xanthine oxidase, while increased inosine may suggest inflammation-induced ATP breakdown, resulting in excessive expression of adenosine deaminase [79]. In addition, the authors found decreased plasma levels of six lysoPCs and alterations in fatty acids, especially decreases in decanoic acid and myristic acid, components of the cell membrane, in OVA-sensitized mice. Decreases were also found for phytosphingosine and sphinganine, which belong to sphingolipid metabolism; for L-tryptophan, which belongs to tryphophan metabolism; and for taurocholic acid, which belongs to bile acid metabolism [79]. In the latter study of this research team, analysis of the lung tissue samples identified several metabolites (L-acetylcarnitine, thromboxane B2, myristic acid, cholic acid fragments, dihydrosphinganine, lysoPC(18:1), palmitic amide, cholic acid, 10-hydroxy docosahexaenoic acid (HDoHE), 5-HETE, lysoPC(15:0), and docosahexaenoic acid) belonging to seven metabolic pathways (i.e., fatty acid metabolism, sphingolipid metabolism, glycerophospholipid metabolism, purine metabolism, tryptophan metabolism, bile acid metabolism and arachidonic acid metabolism) as potential biomarkers of OVA-induced asthma [80]. In plasma of mice exposed to OVA, 25 metabolites, including eight amino acids, nine fatty acids and eight organic acids, were found to be significantly different in comparison with controls. For discrimination between the asthma group and the control, palmitic acid and methionine, followed by pipecolic, lactic, α-ketoglutaric, and linoleic acids, were identified as major potential biomarkers. The results demonstrated a link between asthma and metabolic disturbances in amino acids, fatty acids and intermediate organic acids related to the energy metabolism in the TCA cycle [81]. In the study by Quinn et al., metabolites in the BALF and plasma samples of C57BL6 female mice—divided into controls and three groups according to sensitization status: sensitized/non-challenged mice (OVA/PBS), non-sensitized/challenged mice (PBS/OVA) and sensitized/challenged mice (OVA/OVA)—were analyzed. In OVA/OVA group 4, biological pathways (i.e., arginine and proline, sphingolipid, glycerophospholipid and neurotrophin signaling pathways) were significantly perturbed. In the BALF, 5-aminopentanoate, citrulline and agmatine significantly decreased, and in the plasma, proline decreased, while urea-1-carboxylate level decreased in both the BALF and the plasma. In OVA/ OVA mice, correlations between metabolite changes and lung function were found for increased plasma levels of urea-1-carboxylate that positively correlated with airway hyperresponsiveness, for agmatine that positively correlated with increased eosinophilia, and for ornithine that negatively correlated with airway hyperresponsiveness. The authors concluded that early metabolic changes in the BALF (within 6 h) decreasing over time (as opposed to plasma) may reflect the reaction to the sensitization/challenge at the lung–air interface [82]. The metabolomic analysis of serum of OVA-sensitized animals revealed significant differences in the following metabolites: eicosapentaenoic acid, octadecatrienoic acid, docosapentaenoic acid, otadecadienoic acid, lysoPC (22:6), lysoPC (20:4), lysoPE (0:0/20:2), lysoPC (22:5), lysoPC (15:0), lysoPC (20:2), lysoPE (0:0/22:1) and PC (18:0/0:0) associated with the glycerophospholipid metabolism, fatty acid metabolism, purine metabolism, arginine and proline metabolism, valine, leucine and isoleucine degradation [83]. The OVA-induced asthma model was also used to elucidate the role of polymines promoting the pathogenic potential of inflammatory cells and contributing to airway smooth muscle contraction. The authors demonstrated changes in seven polyamines in lung tissue samples of mice exposed to OVA compared with controls, particularly an increase in levels of putrescine, N1-acetylputrescine and N8-acetylspermidine [84]. In our previous study performed in male guinea pigs, 22 metabolites were significantly altered in the OVA-sensitized group compared with healthy non-sensitized controls. A decrease in levels of total amount of PCs, carnitine, symmetric dimethylarginine (SDMA), ratio of SDMA to total unmodified arginine, and ratio of kynurenine to tryptophan was revealed. On the other hand, we have pointed out an increase in concentrations of tryptophan, taurine, the ratio of methionine sulfoxide to total unmodified methionine and the ratio of total lysoPCs to total PCs as an indicator of phospholipase A2 activity [85].

Naturally occurring equine asthma represents a special animal model of asthma. In recent study, evaluation of lipidomic profile of asthmatic horses revealed significant differences in the plasma lipidomic profile compared with healthy controls. Division of asthmatic horses, based on their BALF cytology, into severe asthma, eosinophilic asthma and neutrophilic asthma groups showed distinct surfactant alterations among the phenotypes, with the most obvious surfactant alterations in phospholipid content and composition found in the severe asthma group, while only mild changes were found in the neutrophilic asthma group [86].

Another animal model of allergic asthma is the model induced by HDM. In BALB/c mice, sensitization with HDM caused significant eosinophilia, neutrophilia and elevations in cytokines including G-CSF, IL-4, IL-5, IL-10, monocyte chemoattractant protein (MCP)-1, macrophage inflammatory protein (MIP)-1α and MIP-1β in the BALF compared with saline controls. Metabolomic investigation of BALF, serum and lung tissue showed distinct compartmental metabolic signatures, which included depleted carbohydrates, increased energy metabolites and consistent losses of sterols and PCs [87]. Furthermore, analyses revealed strong associations between specific metabolic alterations and inflammatory cells and cytokines, linking altered pulmonary metabolism to allergic airway inflammation. For instance, choline, creatine and malate levels negatively correlated with glucose, amino acid, sterol and PC levels; inflammatory cells, IL-4, IL-5, IL-10, IL-13 etc. negatively correlated with sugars, sterols and PCs; and the inflammatory cells and cytokines positively correlated with choline, malate, methyl-hippuric acid and lysyl-arginine levels [87]. In another HDM-induced model of asthma, HDM triggered allergic inflammation, increased mucin MUC5AC in the BALF, and elevated subepithelial collagen deposition and α-smooth muscle actin levels, contributing to airway remodeling. In addition, HDM caused an increase in expression of proteins involved in glycolysis in the lung (likely via IL-1β), confirming that enhanced glycolysis promotes HDM-induced airway hyperresponsiveness and remodeling [88]. Another murine HDM-induced model confirmed histopathological changes associated with allergic response and demonstrated alterations, especially in the compounds linked to the glycerophospholipid and sphingolipid pathways. In HDM-sensitized mice, several lung oxylipins were significantly elevated and calcium channel, G protein-signaling, and mTORC1 signaling pathways were upregulated. Association of allergic sensitization with decreased glycerophospholipid and sphingolipid compounds and increased oxylipins confirmed a role for bioactive lipids in the pathogenesis of allergy and asthma [75].

Another model mimicking Th1/Th17-mediated asthma is the model using PM2.5. In asthma patients, the Th1- and Th17-mediated inflammation characterized by infiltration of Th cells producing interferon (IFN)-γ and IL-17A and neutrophil infiltration is associated with more severe disease and reduced CS sensitivity [89]. Comparison of the effects of sensitization with PM2.5, HDM, and their combination in mice showed that PM2.5 can enhance Th2-mediated allergic sensitization and Th17-associated responses, leading to worsened pulmonary function, and significantly elevated neutrophils, immunoglobulins and Th17-related protein and gene levels in combination the of PM2.5 + HDM compared with HDM only [90,91]. More recently, PM2.5-induced Th1/Th2 imbalance was confirmed in the study by Wang et al. (2019). The authors investigated the metabolic changes in adult female mice associated with exposure of PM2.5 given in three concentrations. Medium and high concentrations of PM2.5 increased levels of IL-4, IL-8, IL-1β, IL-5, IL-13 in BALF, serum IgE, eosinophil count and mucin MUC5AC in the lung, and decreased IFN-γ levels in the lung tissue compared with the controls [92]. In addition, the authors identified 13 asthma-related major metabolites (aspartic acid, butanoic acid, glucose, hexadecanoic acid, idose, inosine, isoleucine, malic acid, octadecanoic acid, octadecenoic acid, serine, uric acid, and valine) that were changed in this asthma model. These metabolites belong to the TCA cycle, purine metabolic pathway, valine, leucine, and isoleucine biosynthesis pathway and alanine, aspartate, and glutamate metabolism pathway. In addition, cytokines IL-4, IL-5, IL-13, IL-1β, and IL-8 correlated negatively with malic acid and positively with aspartic acid, butanoic acid, glucose, hexadecanoic acid, inosine, isoleucine, serine, uric acid and valine levels, while cytokine IFN-γ showed an opposite tendency [92]. A more recent metabolomic study confirmed that exposure of PM2.5 to human bronchial epithelial cells may lead to alteration of numerous metabolic pathways including glycolysis, the citric acid cycle, metabolism of amino acid, and metabolism of lipids. In addition, PM2.5 inhibited the integrin signaling pathway, including downregulating the protein expression of integrins and the phosphorylation of downstream signaling kinases that may affect cell cycle progression, cell metabolism, and apoptosis [93]. Similarly, changes in six pathways, including, e.g., arginine biosynthesis, and valine, leucine and isoleucine biosynthesis involved in the amino acid metabolism and energy metabolism were observed in a rat model of PM2.5-induced model of asthma [94].

**Figure 1:**
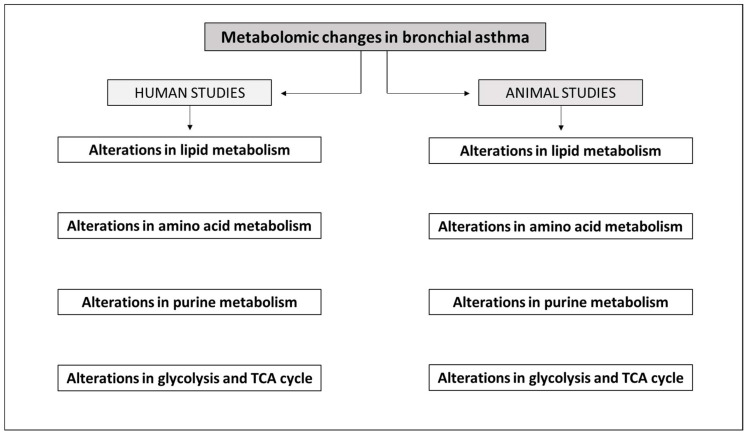
Scheme of alterations of major metabolic pathways demonstrated in human and animal studies. More detailed information is provided in the text and in Table 1 and Table 2. Alterations in lipid metabolism have been demonstrated in both human [29,39,41,43,44,45,51,52,54,55] and animal [75,79,80,81,82,83,85,86,87,93] studies. Alterations in amino acid metabolism have been shown in both human [41,54,58,59,60,61,62,64,65,66,69,72] and animal [79,80,81,82,83,85,87,92,93,94] studies. Alterations in purine metabolism have been confirmed in both human [71,72,73] and animal [79,80,83,92] studies. Alterations in glycolysis and TCA cycle have been demonstrated in both human [55,59,65,67,74,75,76,77] and animal [78,81,87,92,93,94] studies.

The scheme of the above-mentioned alterations of major metabolic pathways demonstrated in both human and animal studies is provided in Figure 1.

### 3.2. Metabolomics Data for Comparison between Obesity-Associated Asthma vs. Healthy Individuals

#### 3.2.1. Human Studies

Other metabolomic studies have demonstrated that there is a close relationship between asthma and obesity. Obesity is an important risk factor for asthma, and asthma associated with obesity is more severe and poorly responsive to CS. Obesity increases airway smooth muscle responses to contractile agents, as detected in human airway smooth muscle cells derived from obese and non-obese subjects [95]. A specific phenotype called obesity-associated asthma has been identified, which is late-onset, rather severe, non-type 2-driven disease, observed mostly in women [96]. Several studies demonstrated that the influence of obesity on asthma is greater in women, while the effect is probably related to the action of estrogens [97,98]. Obesity increased the risk of asthma only in women but not in men [99,100], and asthma severity was more associated with body mass index (BMI) in women [97]. On the other hand, decreasing BMI, e.g., by a plant-based diet, may reduce the asthma symptoms [101].

Obesity is also responsible for alteration of the gut microbiome, contributing to other obesity-related conditions such as insulin resistance and systemic inflammation [102]. Gut bacteria produce a large number of metabolites that may differ in obesity-associated asthma. Shore and Cho described the potential roles for two such classes of metabolites in obesity-related asthma: short-chain fatty acids and bile acids [102].

Maniscalco et al. investigated changes in metabolites in exhaled breath condensate from obese asthmatics, lean asthmatics and obese non-asthmatic subjects. The authors found specific metabolic fingerprints in obese asthmatics that were fully different from lean asthmatics (e.g., higher acetoin, glucose, butyrate, etc. and lower lactate, acetate, formate, etc.), suggesting unique pathophysiologic pathways involved in the pathogenesis of asthma in adult obese subjects [103]. Another study demonstrated that the highest concentrations of glycocholic acid and glycoursodeoxycholic acid can be found in the plasma of obese asthmatics compared with lean asthmatics and healthy lean and obese controls, suggesting the role of bile acid metabolism in the pathogenesis of obesity-associated asthma [104]. Moreover, altered metabolites related to glycolysis and the TCA cycle were found in smooth muscle cells from obese asthmatics compared with non-obese donor cells [105].

#### 3.2.2. Animal Studies

Obesity in mice can be evoked by a high-fat diet (HFD) and/or via modification of the genetic background [106]. However, the type of diet inducing obesity may also influence the resulting metabolomic changes. In obese mice fed with a high-fat/sugar diet or with a very high-fat diet, higher levels of certain metabolites, including complex lipids, free fatty acids, energy metabolites, amino acids, adenosine and NAD pathway members, were observed, while some metabolites were altered in both obese groups compared with controls, and others were different between the diet groups [107]. In addition, the strain of experimental animals may influence the type of immune response and associated metabolic changes. BALB/c mice preferentially developed Th2 responses, with a tendency for liver steatosis associated with differential immune cell composition in the metabolic tissues, while C57BL/6 mice had a tendency to develop Th1 responses and were more susceptible to adiposity, liver inflammation and fibrosis [108].

There have been several studies evaluating the combined obesity + asthma model. For instance, mice with the HFD-induced model of obesity subjected to OVA allergic sensitization showed higher levels of TNFα, IL-5, IL-10 and eosinophil trafficking in BALF than the lean animals [109]. Similarly, higher susceptibility for allergic sensitization was observed in obesity-susceptible AKR mice after OVA sensitization than in animals on a low-fat diet, while serum anti-OVA IgE antibodies and airway eosinophilia correlated positively with the body weight [110]. OVA-induced asthma associated with obesity resulted into an increase in iNOS-expressing macrophages and neutrophils, increased levels of IL-4, IL-9, IL-17A, leptin and IFN-γ in the lung, higher goblet cell hyperplasia, and elevated mast cell influx into the lung and trachea [111]. On the other hand, allergic airway inflammation induced by HDM in mice with HFD-induced obesity was associated with participation of innate lymphoid cells ILC2s and ILC3 [112]. Similarly, HDM-induced asthma accompanied with obesity enhanced neutrophilic airway inflammation and airway hyperresponsiveness linked with higher levels of IL-17A and MIP2 [113].

Comparably to previously mentioned clinical findings of the same authors, obese mice with the HDM-induced model of asthma showed an aggravation in airway inflammation compared with obese controls as well as an increase in bile acid metabolites (β-muricholic acid and tauro-β-muricholic acid) in the serum and lung, confirming alteration of the bile acid metabolism [104]. More recently, the same group of authors, using a similar murine model of obesity-associated asthma, revealed additional interactions between gut levels of amino acid metabolites involved in elastin and collagen synthesis, gut microbiota and lung elastance. Obese mice with allergic airway disease had higher levels of proline and hydroxyproline in the lung, suggesting that changes to structural proteins in the airways and parenchyma contribute to increased lung elastance [114].

### 3.3. Metabolomics Data for Sex Comparisons

#### 3.3.1. Human Studies

As recently reviewed in our article [115], sex-based differences occur already in childhood. In children, the prevalence of asthma is higher in males compared to females, while after puberty, the asthma prevalence reverses to higher values in females. Later, women may suffer from worsening of the asthma symptoms during pregnancy or menstruation. Studies have also revealed that post-menopausal women are more likely to develop the disease or exhibit worsening of its symptoms. This suggests that sex hormone fluctuations play a key role in the pathogenesis of asthma and, therefore, diagnostics and treatment of asthma need a personalized approach [116,117].

Investigation using large-scale targeted metabolomics found that about 33% of metabolites are significantly different between males and females, with differences in steroid metabolism, fatty acids and other lipids, a large fraction of amino acids, oxidative phosphorylation, purine metabolism and gamma-glutamyl dipeptides [118]. Differences in lipid profile between the genders may be associated with risk of cardiovascular diseases or longevity. For instance, low-density lipoprotein (LDL) particle sizes were associated with male longevity, while TG levels, but not LDL particle size, were associated with female longevity [119]. In agreement, another study found in women 19 lipids associated with longevity, including higher levels of ether PC and sphingomyelin species and lower levels of PE and long-chain TG. In addition, a lower ratio of monounsaturated (MUFA) to PUFA fatty acids may suggest that men are more susceptible to oxidative stress [120].

In addition, there have been sex-based differences reported in the immunological response. In males, innate and adaptive immune responses seem to be weaker, resulting in lower susceptibility for developing asthma in adult men than in women, while women display more severe forms of the disease [121]. However, sex disparity already occurs in childhood, when the prevalence of asthma is lower in females than in males and then switches towards females at the onset of puberty. Kelly et al. observed that the interaction of 2-hydroxyglutarate with age appeared to be shifted towards an earlier age in females, mirroring the age of pubertal onset, which typically occurs earlier in females. Further sex differences were also demonstrated for the cholesterol esters [31]. Similar findings of sex differences in metabolome accompanying puberty were more previously published by other authors [122,123]. Zheng et al. found higher urinary levels of citrate, creatinine, hippurate and phenylacetylglutamine and higher plasma levels of PC and unsaturated lipids in girls compared with boys, suggesting sex differences in the metabolome already in asthma adolescents [123]. In another study, plasma lipid profiling revealed significantly higher ceramide in males that was independently associated with age and BMI and lower sphingomyelin in males associated with age. In addition, higher lysophospholipids in males were associated with age, but showed a strong negative association with BMI [122]. In the study comparing metabolites of asthma patients of both sexes, testosterone was negatively related to the inflammatory indicators periostin and IL-5, while estradiol was positively related to the blood eosinophil percentage. Five ceramide (Cer) species and one sphingomyelin species were higher in male than in female patients, with positive correlation between Cer20:0 and IL-5 in males but negative in females [124].

In addition, sex differences were observed in that exacerbations while on ICS treatment were, in females, associated with significant changes in hexadecanedioate and tetradecanedioate, products of fatty acid oxidation, cortisone and cortisol belonging to CS metabolism, mannitol/sorbitol belonging to fructose, mannose, and galactose metabolism, and urea, 5-methylthioadenosine and 1-carboxyethylvaline belonging to polyamine or valine metabolism. On the other hand, only the metabolites associated with CS and fatty acid pathways retained significance in males, who also exhibited higher reduction in these metabolite levels [125].

#### 3.3.2. Animal Studies

In agreement with human studies, metabolomic differences between sexes were also observed in animals. For instance, differences in amino acids, biogenic amines, and acylcarnitines between male and female rats have been described, with higher levels of tyrosine and phenylalanine in males, higher tryptophan in the plasma of females, and significant sex-related changes in the urea cycle [126]. In urine samples or rats, higher levels of sulfate conjugate of m-hydroxyphenylpropionic acid were found in male rats, while higher levels of trimethylamine-N-oxide, N,N′-dimethylglycine, m-hydroxyphenylpropionic acid, N-acetylglycoprotein and cholate were detected in the samples from female animals [127].

Recent studies have demonstrated that sex-specific differences can also be found in asthma models. Hemshekhar et al. evaluated allergen-mediated sex-specific changes in lung protein biomarkers in HDM-sensitized female and male mice as well as in nebulized allergen-sensitized adults. The authors found that from five allergen-increased proteins, eosinophil peroxidase was significantly higher in females compared with males, in both mice and human BALF, but the allergen-induced neutrophil-associated markers S100A8 and S100A9 were significantly higher in BALF of females compared with BALF of males in mice, but significantly higher in males compared with females in humans [128]. In another study, the sex differences in asthma were determined using a murine model of HDM-induced airway inflammation in intact female and male mice, as well as on ovariectomized female mice treated with a physiological dose of 17β-estradiol. The authors found that HDM challenge caused elevated numbers of lung eosinophils, macrophages, and dendritic cells and caused an increase in Th17 cells in the lung and mediastinal lymph nodes only in female mice. However, treatment of ovariectomized mice with physiological levels of estradiol did not influence any of the analyzed cell populations, suggesting that female mice exert more potent innate and adaptive immune responses to HDM challenge, but these effects are not mediated by physiological levels of estradiol [129]. Sex-related differences in immune responses to HDM challenge have been also evaluated in adult mice of two different strains. A female bias was found for an increase in serum HDM-specific IgE in BALB/c mice. In addition, HDM-driven accumulation of neutrophils, eosinophils and macrophages was higher in females compared with males in BALB/c mice. On the other hand, HDM-mediated eosinophil accumulation was more obvious in males compared with females in C57BL/6NJ mice. HDM enhanced a T17-biased response with higher IL-17 levels in female BALB/c mice compared with males, whereas female C57BL/6NJ mice elicited a mixed Th1/Th2-skewed response. Male mice of both strains showed higher levels of specific Th2-skewed cytokines, such as IL-21, IL-25 and IL-9, in response to HDM [130].

In our recent study, sex-based differences in plasma metabolites were already found in healthy animals, as levels of lactate, glucose, and citrate were lower in males than in females. In OVA-sensitized males, higher glucose and lower pyruvate were found compared with healthy male controls. OVA-sensitized females had lower levels of lactate, glucose, alanine, 3-hydroxybutyrate, creatine, pyruvate, and succinate compared with female controls. In OVA-sensitized animals, lactate concentration was lower in males. Generally, data from females (healthy and OVA-sensitized) were more heterogenous. Findings of significant sex differences in plasma concentrations of metabolites found in both healthy and OVA-sensitized animals suggest that sex may influence the metabolism and may thereby contribute to a different clinical picture of asthma in males and females [131].

Combined sensitization with HDM and ozone increased airway hyperreactivity in male mice, but this was not observed in females. Higher airway reactivity in males was accompanied by increased airway inflammation and eosinophilia compared with controls. In addition, combined HDM and ozone exposure significantly elevated glycosphingolipids in both male and female airways, which was also associated with both airway resistance and eosinophilia. However, 15 glycosphingolipid species were increased in females, but only six in males, which was concomitant with significant associations between glycosphingolipids and airway resistance [132].

Mice were also used for development of menopause-associated asthma that was prepared using 4-vinylcyclohexene diepoxide and HDM. Menopause significantly upregulated or downregulated several metabolites in the serum and BALF. Additional metabolomic differences were observed for the Menopause + HDM group compared to Menopause-only or HDM-only groups. HDM challenge in menopausal mice impacted fatty acid elongation and degradation metabolism, purine metabolism, pyrimidine metabolism, pentose phosphate metabolism and biosynthesis of unsaturated fatty acids in the BALF and influenced glycerophospholipid metabolism, arachidonic metabolism, pyrimidine metabolism, and selenocompound metabolism in the menopausal HDM-challenged mice compared with the Menopause-only mice. Other metabolites, including glutamic acid, histamine, uridine, cytosine, cytidine, and acetamide, significantly correlated with total airway resistance [133].

In addition, sex may influence the effect of therapy. For instance, in OVA-induced model of allergic asthma, animals were treated with *Belamcanda chinensis* extract or dexamethasone. The authors identified 39 common differential metabolites among females and males and 37 metabolites that showed opposite regulatory trends in the serum of guinea pigs with asthma and after the treatment, involving several metabolic pathways, including pantothenate and CoA biosynthesis, the arachidonic acid mechanism and glycerophospholipid metabolism, while females were more predisposed to develop asthma changes. The serum levels of metabolites showed distinct sex-specific differences, and the treatment effect of *Belamcanda chinensis* extract also showed sex-specific differences and bidirectional regulation [134].

### 3.4. Metabolomics Data for Age Groups Comparisons

#### 3.4.1. Human Studies

Age is another factor having an impact on asthma course throughout the life. In asthma developing in childhood, Th2-mediated responses and eosinophilia usually dominate, while adult asthma phenotypes are more heterogeneous. Furthermore, the effects of some treatments, e.g., bronchodilators, may be age-dependent [32]. For instance, Kelly et al. investigated the association between age (5–25 years) and bronchodilator response (BDR), the change in airway constriction before and after the administration of short-acting β2-agonist (SABA) that is correlated with asthma control. The authors found that an inverse association between age and BDR in asthmatics may be enhanced with increased 2-hydroxyglutarate, while elevated levels of cholesterol esters, gamma-aminobutyric acid (GABA), and ribothymidine may attenuate the age-associated BDR decline [31]. These findings match well with previous information that 2-hydroxybutyrate regulates production of GABA, having numerous positive effects on the lung functions including bronchorelaxation and reducing neurogenic extravasation and mucus hypersecretion [135], while some cholesterol esters may be associated with mediation of inflammation and immune function [136]. Sordillo et al. found that the indirect effect of age on BDR is mediated through four PC plasmalogens (C36:1 PC and related metabolites) [32]. Plasmalogens have been recognized as mediators of lung function responses and aging, potentially influencing properties of the lung surfactant [137,138]. Results of the study by Sordillo et al. indicated that plasmalogens may contribute to age-related asthma phenotypes, and therefore, they may also serve as a potential pharmacologic target in asthma [32].

Another metabolomic study revealed that metabolic profiles associated with asthma exacerbations while on ICS treatment may change with age from adolescence to adulthood. From the tested metabolites, 38 metabolites showed a significant interaction with age in association with exacerbation, suggesting that these metabolites may be modified over age in asthma cases with exacerbation and could potentially be a target for age-related interventions. Metabolites associated with tryptophan (phenylalanyltryptophan), glycolysis (lactate), fructose metabolism and bile acid metabolism (ursodeoxycholate and glycoursodeoxycholate) were the prominent biomarkers showing a potential interaction with age. Differentiating metabolites may thus serve as biomarkers of ICS response and may highlight metabolic pathways underlying ICS response variability [125].

Summarizing, while the early-life type of asthma is mainly allergy- and Th2-mediated, late-onset asthma may have amixed Th2/Th17 inflammatory profile [139] leading to higher severity and worse response to CS. In addition, higher insensitivity to CS in older asthma patients may be related to mechanisms of lung aging, including persistent inflammation and diminished lung function [140].

#### 3.4.2. Animal Studies

Several animal studies originated to evaluate age-dependent allergen responses later in life. Brandenberger et al. investigated age-dependent changes and Th17 immune response in the HDM-allergen model in male mice. Features of allergic airway disease were more obvious in old compared to young mice; however, AHR was greater in young HDM-treated mice. Only the old mice developed airway neutrophil infiltration and a Th17 immune response upon HDM exposure [141]. Similar results were demonstrated in 3- and 9-month-old female mice exposed to HDM, serving as a model of atopic, perimenopausal female phenotype adult-onset asthma. Both age groups showed potent inflammatory/allergic responses to HDM, but only older HDM-exposed mice had lower lung compliance and increased airway hyperresponsiveness compared with age-matched controls, increases in tissue bronchiolitis, perivasculitis, and BALF neutrophilia relative to their younger counterparts, and a higher extent of immunostaining for IL-4, IL-13, IL-17A and IFN-γ compared with the other groups. Thus, this model showed a difference in inflammatory responses between the young-adult (3-month-old) and middle-aged (9-month-old) female mice, suggesting that neutrophilic more than eosinophilic inflammation is associated with severe asthma symptoms in perimenopausal women [139]. In another asthma model, exposure of older asthmatic rats to PM2.5, a contributor to air pollution, aggravated the asthma, while inflammatory cells and immunoglobulins involved in the immune response, plasma metabolites, and gut microbiota also changed with increasing exposure time [94].

### 3.5. Metabolomics Data Demonstrating Effect of Lifestyle, Diet, and Gut Microbioma

#### 3.5.1. Human Studies

Healthy lifestyle behavior, such as regular physical activity, healthy diet, avoidance of smoking, moderate alcohol intake, and healthy body mass are linked with a decreased risk of metabolic disorders as well as a unique metabolic profile characterized by positive association with beneficial fatty acids and phosphocreatine, and inverse associations with TG, trimethylamine N-oxide, and acylcarnitines [142]. Similarly, regular physical activity leads to beneficial physiological adaptations including metabolic changes such as alterations in energy metabolism (glycolysis, fatty acid and amino acid, oxidation, ketogenesis, TCA cycle), cholesterol, purine, carnitine metabolism, insulin sensitivity, sex hormones biosynthesis, etc., depending on the duration and intensity of the exercise, and type of sport/exercise [143]. Another important factor influencing the risk of asthma is the composition of the diet. For instance, omega-3 PUFA, essential fatty acids from diet necessary for structure and function of membranes, also play an important role in maintaining the balance between gut immunity and the gut microbiota [144]. PUFA are liberated from membrane phospholipids by phospholipase A2 and then converted by lipooxygenase or cyclooxygenase enzymes to various lipid mediators with distinct functions. Omega-3 PUFA, including α-linoleic acid and its metabolites EPA and DHA, exert pro-resolving properties. On the other hand, omega-6 fatty acids are represented by arachidonic acid, a precursor for several pro-inflammatory eicosanoids including leukotrienes, and linoleic acid, a precursor in the synthesis of arachidonic acid. In allergic diseases including asthma, the metabolism of PUFA is dysregulated [49]. For instance, an elevated omega-6-to-omega-3 fatty acid ratio is associated with pro-allergic state, and several studies have shown that dietary intake of omega-3 PUFA may change the composition of the gut microbiome [144]. In children at the age of 3 years, total omega-3, and omega-6 plasma PUFA relative abundances were inversely associated with both asthma and/or recurrent wheeze and allergic sensitization. Dietary PUFA intake was inversely associated with asthma and/or recurrent wheeze [145], while high dietary intake of oleic acid has been associated with asthma [146]. However, the role of omega-3 PUFA in asthma is still unclear and further research in this field is needed. In patients with mild asthma, the metabolomic changes were primarily associated with exogenous metabolites such as higher levels of dietary lipids (linoleic acid, oleic acid, α-linolenic acid etc.) and linoleic acid oxidation products [29], some of which may contribute to Th2 differentiation in asthma [56].

The situation is further complicated by the fact that eosinophils exert diverse functions in inflammation. Eosinophils express 5-lipooxygenase, leading to the production of cysteinyl-leukotrienes contributing to pro-allergic responses, but they may also express 15-lipooxygenase, leading to the production of pro-resolving lipid mediators including lipoxin A4, negatively regulating acute inflammation [49]. In asthma, dysregulation of eosinophils probably leads to dysregulated fatty acid metabolism, with increased cysteinyl-leukotrienes and decreased lipoxin A4 levels [49].

The pathogenesis of chronic inflammatory respiratory diseases including asthma is also related to dysbiosis in microbiota, probably due to dysregulation of host immune response, not only locally in the respiratory tract but also in the gut [147,148]. Microbes generate structural ligands and metabolites that interact with the host and affect the development and progression of chronic respiratory diseases [149]. Even the changes in the nasal microbiota may be related to an increased risk of asthma. While *Corynebacterium* dominates in healthy humans, its relative presence decreases in asthma patients, and the bacterial pathogens *Moraxella*, *Streptococcus* and *Haemophilus* are more frequently observed in the nasal microbiome of patients with asthma compared with healthy humans [150].

Microbiome-derived metabolites have an impact on asthma affecting the immune system through promoting the growth of certain immune cell populations, changing gut diversity and thus enhancing antigenic load. Gut bacteria help to digest polysaccharides, leading to formation of short-chain fatty acids (SCFA) (butyrate, propionate, acetate etc.) that represent energy sources for enterocytes and hepatocytes, but they also act as signaling molecules. Moreover, SCFA may protect against allergic diseases [50]. Some metabolites of dietary fibers, e.g., SCFA that link gut microbiota with asthma, are well-known [151] and, similarly, changes in some gut microbiome products (e.g., phenylacetylglutamine) have been reported during asthma exacerbations [152]. On the other hand, the role of other metabolites, e.g., sphingolipids and their derivatives, should be investigated.

The gut microbiome may modulate immune responses in the lung through microbiomes or their metabolites. Fragments of immune cells or dead bacteria are able to pass through lymphatic circulation to the lung, triggering immune response, while microbiome-derived metabolites such as SCFA may enter the bloodstream and affect the lung immune system [153,154].

#### 3.5.2. Animal Studies

Effects of omega-3 PUFA were also evaluated in animal models of asthma; however, the results were rather controversial. For instance, in a murine OVA-induced model of asthma, Schuster et al. investigated effects of consuming EPA, DHA and EPA-plus-DHA on airway inflammation and hyperresponsiveness. DHA supplementation for 6 weeks led to higher airway resistance, increases in eosinophils and macrophages as well as to increases in IL-6, IL-4 and IL-13 in BALF, while oxylipin production was suppressed [155]. Similarly, dietary omega-3 PUFA impaired sphingolipid metabolism and increased airway inflammation in allergic mice [156]. However, the immune-modulating potential of PUFA depends on the composition of the given PUFA. Supplementation with EPA or the specific combined long-chain polyunsaturated fatty acids (sc)-LCPUFA containing EPA, DHA, γ-linolenic acid and stearidonic acid for 24 days led to distinct fatty acid profiles in plasma, blood cells and lung cells of HDM-sensitized asthmatic mice. In the lung cells of asthmatic mice, arachidonic acid and DHA elevated, while dihomo-γ-linolenic acid (DGLA) lowered. EPA supplementation increased only EPA and docosapentaenoic acid (DPA), while a specific combined dietary supplementation containing n-3 and n-6 LCPUFAs decreased arachidonic acid, increased EPA, DPA and DHA, and reversed the lack of DGLA. The results suggested that in contrast to the EPA supplementation, the combination of n-3 and n-6 LCPUFA restored the LCPUFA profiles in lung tissue of asthmatic mice completely [157]. Interesting also are the findings that resolvinE1, an endogenous lipid mediator derived from omega-3 PUFA, may attenuate airway responsiveness and inflammation in asthmatic mice [158] and promote resolution of inflammation in a model of acute allergic asthma exacerbation [159].

The relationship between the changes in microbiota and airway inflammation has been also confirmed in various animal models of asthma. Similarly to human studies, a diet rich in fiber resulted in higher circulating levels of SCFA in mice that protected against allergic inflammation in the lung, whereas a low-fiber diet decreased levels of SCFA and worsened allergic airway disease [151]. In another model of asthma, a low-fiber diet aggravated inflammation in OVA-induced mice, whereas dietary fiber intake reduced the allergic responses, alleviated allergic signs, decreased eosinophil infiltration and goblet cell metaplasia in the nasal mucosa and lung, lowered serum OVA-specific IgE levels and reduced Th2 cytokines in BALF, but increased Th1 cytokines. Additionally, dietary fiber intake also increased the proportion of *Bacteroidetes* and *Actinobacteria*, decreased *Firmicutes* and *Proteobacteria*, and increased the proportion of probiotic bacteria *Lactobacillus* and *Bifidobacterium* [160]. In an OVA-induced model of eosinophilic asthma in BALB/c mice, several plasma metabolites and gut microbiota biomarkers have been identified. The authors found that plasma metabolites malate and l-dihydroorotate were associated with Th1/Th2 and Treg/Th17 cell balance as well as with genus *Ruminiclostridium 6* of gut microbiota. Similarly, plasma metabolite imidazoleacetic acid was linked with Th1/Th2 cell balance as well as with genus *Ruminiclostridium 6* of gut microbiota. On the other hand, 1,5-anhydro-d-sorbitol was associated with Treg/Th17 cell balance as well as with genus *Ruminiclostridium 6* and genus *Candidatus Arthromitus* [161].

Thus, the experimental results confirmed that dietary fiber intake may promote the balance of Th1/Th2 immunity and attenuate the allergic inflammation associated with intestinal microbiota. Positively influencing lung and/or gut microbiota, several herbal compounds have improved lung functions associated with changes in metabolome in various animal models of asthma [162,163,164].

### 3.6. Metabolomics Data Demonstrating the Effect of Asthma Treatment

#### 3.6.1. Human Studies

Metabolite levels may change not only due to asthma disease itself but also due to delivered treatments. As CS represent a basic treatment in asthma [165], several studies evaluated the effects of CS on the metabolomic profile of patients with asthma. For instance, Reinke et al. found that levels of endogenous steroid metabolites dehydroepiandrosterone sulfate (DHEA-S), cortisone and cortisol significantly decreased in asthmatic patients on maintenance treatment with systemic steroids [29], reflecting hypothalamus–pituitary–adrenal axis suppression [166]. However, DHEA-S also decreased in patients on ICS alone, demonstrating a systemic effect of ICS treatment [29]. In agreement with these findings, metabolomic profiling in the observational study of 4-year low-dose ICS treatment in asthma patients revealed adrenal suppression associated with increased risk of fatigue and anemia [167].

A study by Daley-Yates et al. compared the metabolomics/lipidomics profile after three different ICS treatments (fluticasone furoate, fluticasone proprionate, budesonide) in 54 asthmatics. The authors pointed out that metabolomic changes (reduced adrenal steroids, particularly glucuronide metabolites of cortisol and cortisone and pregnenolone metabolite DHEA-S, increased amino acids and glycolytic intermediates, decreased fatty acid β-oxidation and branched-chain amino acids) were seen predominantly at the highest/supratherapeutic doses of ICS [168]. In addition, prolylhydroxyproline, pipecolate and N-palmitoyltaurine correlated significantly with ICS, and were further shifted in individuals treated with oral CS [29]. Ceramides, sphingomyelin and four free fatty acids (myristoic, palmitoleic, eicosenoic and dihomo-gamma-linolenic acid) were positively associated with the dose of ICS, while oleoylethanolamide increased with asthma severity independently of CS treatment [29]. The study indicated that metabolic changes associated with high-dose medication were more pronounced than metabolic changes related to the disease itself. Nevertheless, because all individuals with moderate or severe asthma in the study by Reinke et al. [29] were treated with CS, authors were not able to fully distinguish between the effects of disease severity and treatment.

However, there is a significant portion of asthma patients who exert CS resistance [165]. It is supposed that there are multiple factors responsible for CS resistance, such as oxidative stress and smoking, as well as distinct molecular mechanisms, including suppressed nuclear translocation of glucocorticoid receptor (GR)α after binding CS, higher expression of GRβ, elevated secretion of macrophage migration inhibitory factor, competition with the transcription factor activator protein-1 or decreased expression of histone deacetylase-2 [169]. In addition, resistance to CS may originate from different pathophysiological features, and the type of inflammation in distinct endotypes of asthma as a response to CS in non-eosinophilic inflammation is much lower than in eosinophilic inflammation [170]. In children with severe asthma, metabolic alterations linked with oxidative stress-related pathways, i.e., the glycine, serine and threonine metabolism pathway and the N-acylethanolamine and N-acyltransferase pathway, were observed, suggesting that oxidative stress-associated changes may contribute to CS insensitivity in severe asthma [171]. High-resolution metabolomics of urine samples of children showed significantly different levels in five metabolites between CS responders and CS non-responders, including 3,6-dihydronicotinic acid, 3-methoxy-4-hydroxyphenyl(ethylene)glycol, 3,4-dihydroxy-phenylalanine, γ-glutamylcysteine and cysteine-glycine, and reduced flavin mononucleotide, suggesting that tyrosine metabolism, degradation of aromatic compounds and glutathione metabolism are involved in CS resistance [172].

Nevertheless, other asthma treatments may also influence the metabolism [173]. For instance, sustained β2-receptor activation due to administration of albuterol in normal healthy subjects enhanced lactate production and altered aerobic glycolysis, gluconeogenesis and free fatty acid metabolism. On the other hand, during asthma control with albuterol, the arachidonic acid metabolism and linoleic acid metabolic pathways were altered, and two metabolites (monoHETE_0863 and sphingosine-1-phosphate) were modified before and after asthma control [174]. Significant metabolic changes were also demonstrated for the combination of inhaled budesonide and salbutamol in asthmatic children with acute exacerbation. The authors revealed 22 different metabolites in the serum and 21 metabolites in the urine of budesonide + salbutamol treated asthmatic children compared with asthmatic children treated with a placebo, suggesting reprogramming in seven metabolic pathways: arginine and proline metabolism; taurine and hypotaurine metabolism; glycine, serine and threonine metabolism; glyoxylate and dicarboxylate metabolism; methane metabolism; citrate cycle; and pyruvate metabolism [175]. On the other hand, treatment of adult asthmatics with inhaled fluticasone furoate/vilanterol trifenatate was accompanied by only subtle systemic metabolomic and lipidomic changes [176].

#### 3.6.2. Animal Studies

The effects of various asthma treatments on the metabolome were also evaluated in several animal studies. For instance, in an OVA-induced murine model of allergic asthma, the metabolic profiling of BALF revealed alterations of energy metabolism, with increases of lactate, malate and creatinine, and reductions in carbohydrates, such as mannose, galactose and arabinose. the lipid and sterol metabolisms were also affected with declines in PCs, diglycerides, TGs, cholesterol, cortol and cholic acid. Dexamethasone given 1 h before each OVA challenge reversed many key metabolite changes, but was ineffective in repressing lactate, malate and creatinine, and induced additional metabolite changes. Dexamethasone administration resulted in increases in fatty acids, including steridonic acid, eicosapentaenoic acid and petroselinic acid, as well as various monoglycerides, diglycerides, TGs, sphingomyelin and lysoPCs. In addition, dexamethasone elevated the amino acids N,N-dimethyl-tyramine and N-acetyl-tyrosine and reduced 2-oxoarginine, a metabolite of arginine catabolism [177].

In another murine model of allergic asthma induced by environmental aeroallergen HDM, BALF, lung tissues and blood were investigated. Comparison of animals with asthma model vs. controls showed changes in metabolome, including a decrease in carbohydrate metabolites (glucose, mannose, galactose), increased energy metabolites and consistent reductions in sterols and PCs [177]. The authors also identified an involvement of the glucose–alanine metabolic pathway in inflamed lungs, characterized by declined glucose upstream and a corresponding increase in downstream alanine and by-products, urea, L-glutamate and glutamine. The specific metabolic alterations were associated with changes in inflammatory cells and cytokines, linking altered pulmonary metabolism to allergic airway inflammation. Administration of the corticosteroid prednisolone was effective against HDM-induced airway eosinophilia but did not suppress neutrophilic inflammation [87]. However, compared with dexamethasone, prednisolone showed weaker reversal effects against HDM-induced metabolic alterations, probably due to unresolved neutrophilic inflammation [177]. In addition, prednisolone in HDM-sensitized animals reversed changes in HDM-induced metabolites, choline, creatine and malate and promoted increases in amino acids, homoserine, tyrosine, methionine and thymidine [87].

In guinea pigs with OVA-induced allergic asthma model, dexamethasone alleviated lung inflammation, edema and airway fibrosis, decreased nuclear factor (NF)-κB expression and lowered IL-4, IL-5, IL-13 and IgE levels in serum. In addition, dexamethasone downregulated leukotriene B4, a pro-inflammatory mediator, that was increased in asthma animals compared with controls. On the other hand, OVA-induced decreases in lysoPC (18:0) and lysoPC (20:3), anti-inflammatory mediators, were upregulated by dexamethasone [178].

In another study carried out on OVA-sensitized mice, the effects of dexamethasone were compared to those of Modified Kushen Gancao Formula (mKG), derived from traditional Chinese herbal medicine. Dexamethasone and mKG showed a similar anti-asthma effect on the lung histopathology. Both treatments also had a similar effect on fatty acid metabolism and ameliorated abnormal changes in the arachidonic acid, sphingolipid and glycerophospholipid metabolisms (except that there was no effect of mKG on lysoPC (15:0)). In addition, mKG and dexamethasone reversed a decrease in uric acid and an increase in inosine, suggesting an effect of the treatments against purine metabolism disorder in the development of allergic asthma. Both treatments also upregulated L-tryptophan that was suppressed by OVA. Treatment with mKG upregulated the levels of cholic acid and taurocholic acid and thereby improved the dysfunction of bile acid metabolism induced by OVA [80].

The metabolic changes in OVA-induced asthma mice were significantly reversed by surfactant protein A (SPA), i.e., disturbances in purine, glycerophospholipid, fatty acid, phenylalanine, fatty acyls, arginine and proline, valine, leucine and isoleucine, glycine, serine and threonine and amino acid biosynthesis metabolisms were alleviated after SPA treatment [83].

Several fundamental metabolomic studies performed in humans and in murine and guinea pig animal models are provided in Table 1 and Table 2.

**Table 1:** Human metabolomic studies of bronchial asthma.

Asthma Population; Reference	Participants	Metabolomic Analysis; Samples	Main Results
Adults [52]	18 Non-steroid treated asthma, 20 steroid-treated asthma, 13 controls	HPLC-QTOF-MS; BALF	↑ LPC, TG, PC, PG, PS, SM in non-steroid treated asthma vs. controls; no difference between steroid-treated asthma vs. controls
Adults [55]	57 Asthma	Targeted SPME/GCxGC-TOF/MS; urine	Lipid peroxidation metabolites associated with asthma severity, lung function and eosinophilic inflammation
Adults [29]	54 Asthma/22 controls	Untargeted LC-MS/ Targeted LC/MS; serum	↑ Ceramide, sphingomyelin, hexosylceramide, LTE4 in asthma vs. healthy; ↓ 14,15- DiHETE, 19,20- DiHDPA in asthma vs. healthy
Adults [51]	15 Asthma, 15 controls	UHPLC-QTOF-MS; serum	↑ 5(S)-HETE, 8(S)-HETE, 11(S)-HETE, 12(S)-HETE, 15(S)-HETE, 15(S)-HEPE, ProstGA2, ProstG B2, ProstG F1a, ProstG F2a, ProstG J2, 15-keto-ProstG F2a in asthma vs. control, ↓ palmitic acid, lauric acid in asthma vs. controls
Adults [103]	25 Obese asthma, 30 obese non-asthma, 30 lean asthma	Untargeted NMR; EBC	Respiratory metabolic profile in obese asthmatics divergent from other patient groups; differences in methane, pyruvate, and glyoxylate and dicarboxylate pathways
Adults [44]	13 Eosinophilic asthma (EA), 16 non-eosinophilic asthma (NEA), 15 healthy controls	Untargeted UPLC-MS; serum	Changes in glycerophospholipid, retinol, sphingolipid, galactose and inositol phosphate metabolisms for EA vs. NEA
Adults [39]	33 Asthma, 28 healthy controls	LC-MS/MS-based lipidomics; plasma	↑ PE (18:1p/22:6), PE (20:0/18:1), PE (38:1), SM (d18:1/18:1), TG (16:0/16:0/18:1) in asthma vs. healthy; ↓ PI (16:0/20:4), TG (17:0/18:1/18:1), PG (44:0), ceramide (d16:0/27:2), LPC (22:4) in asthma vs. controls
Children [179]	50 asthma, 49 healthy controls	Targeted LC-MS; serum	↓ Ascorbic acid, 2-isopropylmalic acid, shikimate-3-phosphate, 6-phospho-D-gluconate, and reduced glutathione in asthma vs. controls
Children [180]	380 children with asthma	Targeted LC-MS; plasma	Glycerophospholipid, linoleic acid, and pyrimidine metabolisms associated with AHR, and pre- and postbronchodilator FEV1/FVC
Children [181]	30 children with asthma, 30 controls	NMR; urine	↓ 1-Methylnicotinamide and allantoin in asthma vs. controls
Children [64]	13 asthma, 17 healthy controls	LC-MS/MS; serum	↑ L-arginine, Β-alanine, Ƴ-amino-N-butyric acid, L-histidine, hydroxy-L-proline in asthma vs. controls; ↓ D,L-Β-Aminoisobutyric acid, taurine, L-tryptophan, L-valine in asthma vs. controls
Children [77]	92 children with asthma, 73 controls	NMR; EBC	↑ Lactate, formate, butyric acid, isobutyrate in asthma

Notes: ↑: increase, ↓: decrease. Abbreviations: AHR: airway hyperreactivity, BALF: bronchoalveolar lavage fluid, COPD: chronic obstructive pulmonary disease, DiHETE: dihydroxyeicosatetraenoic acid, DiHDPA: dihydroxydocosapentaenoic acid, EA: eosinophilic asthma, EBC: exhaled breath condensate, FEV1/FVC: forced expiratory volume in the first second/forced vital capacity, GC-MS: gas chromatography-mass spectrometry, HEPE: hydroxyeicosapentaenoic acid, HETE: hydroxyeicosatetraenoic acid, HPLC: high-performance liquid chromatography, LC-MS: liquid chromatography-mass spectroscopy, LPC: lysophosphatidylcholine, LTE4: leukotriene E4, NEA: non-eosinophilic asthma, NMR: nuclear magnetic resonance, PC: phosphatidylcholine, PE: phosphatidylethanolamine, PG: phosphatidylglycerol, PI: phosphatidylinositol, ProstG: prostaglandin, PS: phosphatidylserine, SM: sphingomyelin, SPME/GCxGC-TOF/MS: solid phase microextraction with two-dimensional gas chromatography and time-of-flight mass spectroscopy, QTOF MS: quadrupole time-of-flight mass spectrometry, TG: triglyceride, UHPLC-QTOF-MS: ultra-high performance liquid chromatography quadrupole time-of-flight mass spectrometry, UPLC-MS: ultra-performance liquid chromatography-mass spectrometry.

**Table 2:** Metabolomic studies of bronchial asthma in murine and guinea pig animal models.

Model of Asthma; Reference	Animals (Groups)	Metabolomic Analysis; Samples	Main Results
OVA-induced asthma [78]	Dunkin-Hartley female guinea pigs (controls, controls treated with DEX, OVA-sensitized, OVA-sensitized + challenged, sensitized + challenged treated with DEX)	NMR; urine	Urine metabolites correlated with airway dysfunction in asthma model
OVA-induced asthma [177]	BALB/c female mice (OVA-induced asthma, OVA-induced asthma treated with DEX, controls)	GC-MS, LC-MS; BALF	Alterations of energy metabolism, carbohydrate, lipid and sterol metabolisms in asthma model; partial reverse by DEX, but DEX ineffective in decreasing lactate, malate and creatinine
OVA-induced asthma [79]	BALB/c female mice (OVA-induced asthma model, controls)	Untargeted UPLC-Q-TOF/MS; plasma	Changes in purine, sphingolipid, glycerophospholipid, FAs, tryptophan and bile acid biosynthesis metabolism in asthma model
OVA-induced asthma [83]	BALB/c female mice (OVA-induced asthma, OVA-induced asthma treated with SPA, controls)	Untargeted UPLC-Q-TOF-MS; serum	Changed 32 metabolites in 9 metabolic pathways in asthma model; significant reverse after SPA treatment
OVA-induced asthma [82]	C57BL/6 female mice (OVA sensitized and/or challenged, controls)	Untargeted HPLC-TOF/MS, Targeted HPLC-MS; BALF and plasma	Changes in sphingolipid, glycerophospholipid, arginine and proline metabolisms, and neurotrophin signaling pathway in asthma model; AHR correlated with urea-1-carboxylate and ornithine; lung eosinophilia correlated with agmatine
OVA-induced asthma [81]	C57BL/6 female mice (OVA sensitized, controls)	GC-MS; plasma	Changes in 25 metabolites, including eight AAs, nine FAs and eight OAs; most significant changes in palmitic acid, methionine, pipecolic, lactic, α-ketoglutaric and linoleic acids
OVA-induced asthma [80]	BALB/c female mice (OVA-induced asthma, OVA-induced asthma treated with DEX or mKG, controls	Untargeted UPLC-Q-TOF/MS; lung tissue and plasma	Changes in 24 metabolites including myristic acid, sphinganine, and lysoPC in lung and plasma of asthma model; l-acetylcarnitine, thromboxane B2, 10-HDoHE, and 5-HETE as potential biomarkers; mKG and DEX influenced the biomarkers; DEX less effective
OVA-induced asthma [178]	Hartley male guinea pigs (OVA-sensitized, OVA-sensitized and treated with DEX or AST, controls)	UPLC-ESI-QTOF/MS; serum	AST therapy restored phospholipid, sphingolipid, purine, AAs and epinephrine levels back to normal control level; AST could alter the sphingolipid metabolism
OVA-induced asthma [85]	TRIK male guinea pigs (OVA-sensitized, controls)	Targeted UPLC/MS; plasma	Changes in 22 metabolites, ↓ PC, carnitine, dimethylarginine, dimethylarginine/arginine ratio, kynurenine/tryptophan ratio, ↑ tryptophan, taurine and methionine sulfoxide/methionine ratio in asthma model
OVA-induced asthma [134]	Guinea pigs (controls, OVA-sensitized, OVA-sensitized and treated by DEX or BCE)	UPLC-MS; serum and BALF	Sex-based differences in 39 metabolites, changes in 37 metabolites in asthma animals involving 17 metabolic pathways; BCE improved nerve and energy metabolism; sex-specific differences for BCE
HDM + ozone- induced-induced asthma [132]	BALB/c female and male mice (HDM + ozone-sensitized, controls)	LC-MS/MS; BALF and lung tissue	↑ glycosphingolipids associated with ↑ AHR and airway inflammation in males and females, but more severe in females
PM2.5-induced asthma [92]	BALB/c female mice (controls, three concentrations of PM_2.5_)	GC-MS; lung tissue	Medium and high concentrations of PM2.5-induced asthma linked with changes in 13 metabolites associated with oxidative stress and metabolism

Notes: ↑: increase, ↓: decrease. Abbreviations: AAs: amino acids, AHR: airway hyperreactivity, AST: acupoint sticking therapy, BALF: bronchoalveolar lavage fluid, BCE: *Belamcanda chinensis* extract, DEX: dexamethasone, FAs: fatty acids, GC/MS: gas chromatography/mass spectrometry, GSTP: glutathione-S-transferase P, HDM: house dust mite, 10-HDoHE: 10-hydroxy docosahexaenoic acid, 5-HETE: 5-hydroxyeicosatetraenoic acid, HPLC-MS: high-performance liquid chromatography-mass spectrometry, HPLC-TOF/MS: high-performance liquid chromatography coupled with quadrupole time-of-flight mass spectrometry, LC/MS: liquid-chromatography/mass spectrometry, LC-Q-TOF-MS: liquid-chromatography time-of-flight mass spectrometry, mKG: modified Kushen Gancao Formula derived from traditional Chinese herbal medicines, NMR: nuclear magnetic resonance, OAs: organic acids, OVA: ovalbumin, PC: phosphatidylcholine, PM2.5: fine particular matter, SPA: surfactant protein A, UPLC-Q-TOF/MS: ultra-high-performance liquid chromatography coupled with quadrupole time-of-flight mass spectrometry, UPLC-ESI-QTOF/MS: ultra-performance liquid chromatography combined with electrospray ionization quadrupole time-of-flight mass spectrometry.

## 4. Translational Value and Limitations of Animal Models of Asthma

Various animal models of asthma have been performed, using distinct protocols to mimic human asthma. Acute models of asthma may be successfully used to elucidate the key pathophysiological features of asthma, including cell-mediated lung inflammation, disease mediators, T-cells and eosinophils and their role in asthma, etc. [182]. In order to resemble human allergic asthma, protocols of preparing asthma model involve both allergen sensitization and allergen challenge. Sensitization is traditionally performed by intraperitoneal and subcutaneous routes, but also by intranasal instillation of allergens; challenges with allergens are performed through aerosol, intranasal or intratracheal instillation [182]. Chronic models are not as common as acute models. However, after several weeks of systematical sensitization of animals, chronic changes including epithelial hypertrophy, goblet cell metaplasia, subepithelial fibrosis and smooth muscle hyperplasia may be achieved [183].

Models induced by OVA, HDM and PM2.5 in mice or guinea pigs are the most commonly used models in the asthma pre-clinical research; however, there are some limitations for their translational potential. First, these models evoke responses to only one specific allergen, while humans are constantly exposed to a plethora of various allergens possessing distinct antigen potency. Second, airway hyperreactivity and airway inflammation may, in animals, resolve within a few weeks after the final allergen challenge, while in humans, the inflammation persists and the repeated provocation by the allergen induces a recurrence of symptoms [184]. There are also specific limitations for the used triggering factor. For instance, OVA, a protein from chicken egg white, is a traditionally used allergen in animal models; however, it does not induce intensive allergic airway inflammation in humans. In addition, the prolonged exposure to OVA may lead to tolerance [182,185]. On the other hand, the benefits of OVA result from the fact that this substance is efficient and cheap, and the immune response to OVA is well-characterized. In order to potentiate the immune response to OVA, adjuvants may be added, e.g., Al(OH)_3_, to aggravate an antigen-specific Th2-mediated inflammatory response [182,185]. Administration of HDM containing heterogenic extract of *Dermatophagoides* species and products plausibly mimics the clinical situation, as 50–85% of asthmatics are HDM-allergic and have elevated HDM-specific IgE antibodies [35]. Protein products of *Dermatophagoides* act through the TLR-4 receptor signaling pathway, resulting in Th2-mediated allergic inflammation; however, an increase in the serum levels of a specific IgE antibody is not so obvious in OVA models or human asthma [35]. On the other hand, repeated exposure to HDM leads to remodeling of the airways, increased mucous cell density and airway hyperreactivity, but, compared to the OVA model, the remodeling changes remain relatively stable even after discontinuation of HDM exposure [186]. Furthermore, a combination of HDM and OVA exposures may be favorable, as the inflammatory response may be augmented and HDM may prevent the tolerance evoked by prolonged OVA administration [187,188].

Discrepancies and variations among the used study protocols, animal species and allergens cause the results from animal studies performed on small animals to be sometimes contradictory or difficult for interpretation and clinical translation. Therefore, there is an increasing interest in the naturally occurring equine asthma, which may bring valuable information for asthma researchers [189]. In comparison to the above mentioned animal models, where changes mimicking human asthma require artificial sensitization and challenge, asthma in horses may originate due to triggering factors similar to those in humans, such as exposure to environmental dust or infectious agents. In addition, features of equine asthma, including mild to severe chronic respiratory signs, inflammatory airway disease and recurrent airway obstruction, closely resemble human asthma [190]. Several endotypes and phenotypes of equine asthma have even been recognized [189,191]. To enhance laboratory verification of equine asthma or to differentiate the asthma endotypes and phenotypes, several metabolomic studies have been recently published [86,192,193], demonstrating significant changes in lipid metabolites.

Considering the mentioned limitations, animal models represent a valuable tool in asthma research. Data obtained from asthma models performed in mice, guinea pigs and horses help to elucidate the pathophysiological mechanisms of asthma and may be useful in identification of asthma-associated metabolomic markers, under the umbrella of the “One Health” concept in future [194].

## 5. Conclusions

Clinical signs of asthma result from a broad spectrum of inflammatory, immunological, biochemical and metabolic perturbations. The majority of these alterations have been elucidated by means of animal models. Further information is brought by novel “omics” methods. One of them, metabolomics, has a huge potential to provide a detailed insight to asthma pathophysiology and find specific biomarkers helpful in diagnostic process and management of asthma. As demonstrated in the above-mentioned studies, metabolomic changes in the animal studies resemble, to a large extent, the changes found in human patients with asthma. Thus, despite the limitations of animal modeling in asthma, the results suggest that pre-clinical testing and metabolomic analysis of animal samples may, together with metabolomic analysis of human samples, contribute to a novel way of personalized treatment of asthma patients.

## Data Availability

Data is contained within the article.
